# Effect of Temperature on Material Removal Rate During Shear-Thickening Polishing

**DOI:** 10.3390/ma18092033

**Published:** 2025-04-29

**Authors:** Zhong Yu, Jiahuan Wang, Jiahui Du, Lanying Shao, Binghai Lyu

**Affiliations:** College of Mechanical Engineering, Zhejiang University of Technology, Hangzhou 310023, China; 211122020090@zjut.edu.cn (Z.Y.); 221122020476@zjut.edu.cn (J.D.); shaolanying1997@163.com (L.S.)

**Keywords:** shear-thickening polishing, temperature, high-speed video, material removal rate

## Abstract

Shear-thickening polishing (STP) technology achieves efficient processing by modulating the non-Newtonian properties of the slurry, while temperature has an important effect on its rheological behavior. To reveal the effect of temperature on material removal rate (MRR) during the shear-thickening polishing process, this study measured the rheological profiles of the shear-thickening polishing slurry (STPS) at different temperatures and observed the rheological behavior using a high-speed video camera, as well as monitored the changes in the polishing force exerted on the workpieces, MRR, and the surface roughness. Experimental data show that the peak viscosity of the slurry in the shear-thickening state decreases from 0.81 Pa·s to 0.49 Pa·s as the temperature increases from 30 °C to 50 °C. High-speed video observations show that the wavy solid layer in the thickening area diminishes with increasing temperature, the distribution area shrinking, and nearly vanishing at 50 °C. When the temperature rises from 30 °C to 40 °C, the average polishing force at 30 min decreases from 25.3 N to 22.6 N by 10.6%. MRR decreases from 33.5 nm/min to 7.9 nm/min by 75.5%. The decrease in MRR is much greater than the polishing force. This study provides an experimental basis for the effect of temperature on STP.

## 1. Introduction

Curved-surface components with fine surface quality play a crucial role in determining the performance of high-end equipment and systems involving aerospace equipment, biomedical implants, and optical system. As a core process in ultra-precision machining, polishing not only achieves nanometer-scale surface accuracy but also effectively removes subsurface defects and precisely controls the surface roughness parameters of curved surfaces, thereby significantly enhancing the overall performance and longevity of the components. To address the technical challenges of machining complex curved surfaces, advanced polishing techniques such as airbag polishing [1], abrasive flow polishing [2], and magnetorheological polishing [3,4] have been developed in the past decades, and have achieved successful applications. However, polishing process with high quality, high efficiency and low cost is still expected.

**Figure 1:**
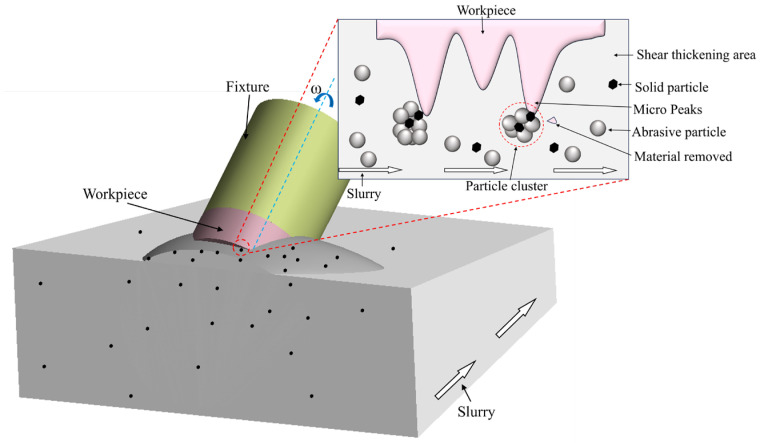
Schematic diagram illustrating the principle of microscopic material removal in the STP process.

A novel STP procedure based on the characteristics of non-Newtonian fluids has been developed in response to the ongoing rise in the machined surface’s curvature [5,6,7,8,9,10]. Li and Lyu et al. [11] first introduced this concept in 2015, incorporating a shear-thickening fluid (STF) and an abrasive composite system into the polishing process. By controlling the viscosity variation of the slurry under high-speed shear, this method enables efficient material removal with low equipment requirements and low cost. Among them, STP is a precision machining technology based on the properties of non-Newtonian fluids. Its core principle utilizes the unique rheological behavior of STPS. STPS is a low-viscosity slurry under normal conditions, but when subjected to high-speed shear (such as mechanical stirring or impact), its viscosity rises rapidly and even exhibits solid-like properties. During the polishing process, micron or nano-scale abrasive particles are dispersed in STF to form a polishing medium. When the STPS and the workpiece surface move relative to each other, the shear rate in the contact area rises sharply, triggering a sudden increase in slurry viscosity. This instantaneous thickening effect causes the abrasive particles to produce directional micro-cutting under high pressure. At the same time, the thickened slurry forms a flexible “elastic cutting tool” that effectively removes the microscopic rough peaks on the workpiece surface, as shown in Figure 1. Lyu et al. [12] found that polishing speed has the most significant influence on polishing performance. Increasing the speed enhances the shear rate and strengthens the shear-thickening effect, thereby significantly improving the MRR and surface roughness. The effect of abrasive concentration on polishing performance is similar to that of speed, while abrasive size has little impact on surface roughness. However, a decrease in abrasive size results in an increased MRR. Wang et al. [13] compared three recently developed polishing techniques suitable for Ti-6Al-4V biomaterials: STP, chemically enhanced STP (C-STP), and electrolytically enhanced STP (E-STP). The study revealed that after STP, the surface roughness Sa was approximately 13.8 nm, with an MRR of 54.3 nm/min. In comparison, C-STP achieved nearly half the surface roughness of STP and exhibited an MRR approximately 2.1 times higher than that of conventional STP. Compared with STP, which does not cause changes in surface composition, a 136 nm thick Beilby layer was observed during C-STP, indicating a lower friction coefficient. E-STP demonstrated intermittent improvements, achieving a surface roughness of 10.2 nm and an MRR 1.6 times that of conventional STP. These findings suggest that the application of STP can be further enhanced through chemical modifications. He et al. [14] investigated STP using a textured hollow polishing tool. Through a combination of simulations and experiments, they obtained a relative error of 8.36% between experimental and theoretical values and successfully reduced the surface roughness of CaF_2_ to 5.9 nm under optimized conditions. Ma et al. [15] established an MRR model for the magnetic STP process, demonstrating its effectiveness by reducing the surface roughness Sa of aluminum workpieces to 79.0 nm, improving roughness by over 77%, with an average theoretical-experimental error of 4.1%. Span [16] found that adding coarse abrasives during the polishing process can obtain better surface morphology, while fine abrasives are difficult to effectively remove deep surface pits. Nguyen et al. [17] demonstrated that the inclination angle between the workpiece and the flow field significantly influences the post-polishing surface quality, with an optimal range of 5° to 15°; deviations beyond this range lead to inferior surface morphology.

The above studies assume that the rheological properties of the slurry are stable during STP. However, as non-Newtonian fluids, STPS exhibit significant fluctuations in rheological properties with temperature variations [18,19,20]. The STPS used in this article contains polyhydroxy polymer solid phase particles. The increase in temperature will cause these particles to denature, thereby affecting their shear-thickening properties [21,22]. Tian [23] studied the change in viscosity with shear rate in the range of 20 °C to 60 °C. The results showed that higher temperature will increase the critical shear rate and reduce the shear-thickening rate. Li [24] investigated the discontinuous shear-thickening phenomenon of silica nanoparticle suspensions and found that the phenomenon is more significant below 40 °C. Li [25] conducted pull-out tests on STF/Kevlar fabric at various temperatures ranging from 20 °C to 50 °C. The results demonstrated that at 50 °C, STF containing more hydroxyl groups and longer molecular chains mitigated the adverse effects of temperature increase on the friction performance of the yarn, reducing the degradation from 88.94% to 49.23%. Existing studies have clearly shown that temperature fluctuations can significantly change the rheological properties of shear-thickening systems. However, nothing is known about how dynamic temperature changes affect STP’s polishing force and material removal effectiveness.

This study aims to investigate the effect of temperature on MRR during STP. Quartz glass with low linear expansion coefficient, high temperature resistance and excellent chemical stability is used as the polishing object. The variations in rheological properties at different temperatures and their effect on polishing force and MRR are analyzed.

## 2. Experiment

### 2.1. Experimental Setup and Procedures

**Figure 2:**
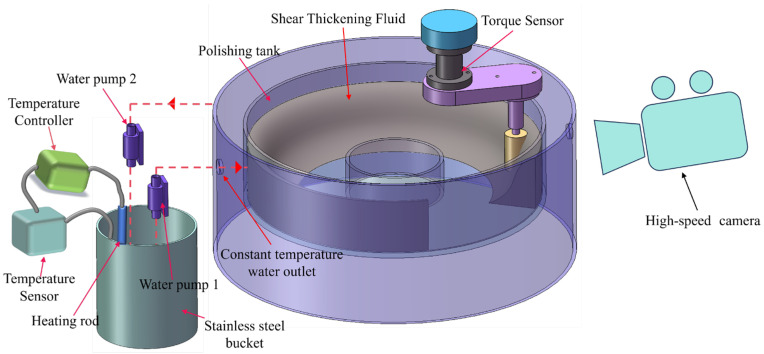
Schematic diagram of temperature controlled polishing device.

This experiment aims to measure the polishing force exerted by the STPS on the workpiece at varying temperatures. Therefore, a temperature control system is designed for the experimental device to ensure that the STPS operates stably at the set temperature. Before measuring the polishing force, a high-speed camera captures the slurry deformation during STP, enabling analysis of the dynamic evolution of the shear-thickening phenomenon. As illustrated in Figure 2, the high-speed camera records the slurry morphology at the interface between the fixture and the STPS, capturing structural changes under shear and providing a theoretical foundation for subsequent polishing force measurements.

As illustrated in Figure 2, the polishing force measurement device consists of a temperature control system and a torque measurement unit. The temperature control system consists of a stainless steel barrel and an internal heating element. The system maintains a constant water temperature by precisely adjusting the power of the heating element. Water pump 1 extracts temperature-controlled water from the barrel and directs it through a pipeline to the polishing disc wall for spraying. The arrows in the figure denote the direction of water flow. Heat conduction adjusts the temperature of the STPS in the polishing tank. Under constant temperature conditions, the STPS rotates with the polishing disc, generating relative motion with the stationary workpiece, forming a shear rate gradient, causing the workpiece surface to be stressed and achieving material removal. The torque sensor measures the torque exerted by the STPS on the workpiece in real time and calculates the actual polishing force using the lever arm principle. Water pump 2 is responsible for pumping the water in the polishing tank back into the heating barrel through the reflux port to achieve circulating heating of the water.

The experimental device combines a temperature-controlled system, high-speed video, and precise force measurement, which can systematically study the rheological properties of STPS at different temperatures and their polishing effects on workpieces, providing experimental support for the optimization of STP technology.

**Figure 3:**
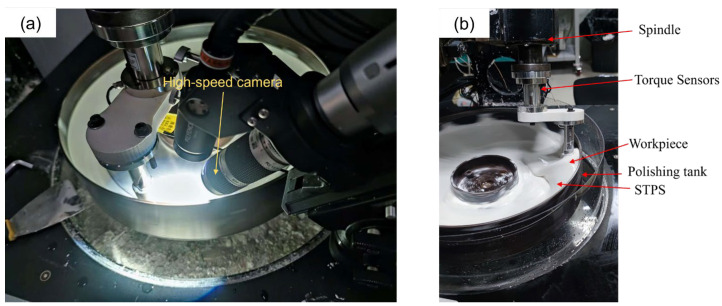
Experimental setup. (**a**) High-speed camera setup, (**b**) polishing force measurement setup.

The experimental setup is shown in Figure 3a, which depicts the experimental setup for shooting shear-thickening rheological behavior with a high-speed camera. Figure 3b illustrates the polishing force measurement device. In order to more clearly capture the typical thickening characteristics when STPS impacts the workpiece surface, the image of the change in the STPS when the front of the workpiece is impacted by the STPS is taken. The quartz glass is placed at the bottom of the workpiece. To ensure that it is completely immersed in the STPS, the depth of the workpiece immersed in the STPS is 25 mm. When the STPS undergoes the shear-thickening effect, the high-speed camera records the shear-thickening image, and the torque sensor collects data and calculates the actual polishing force.

When the temperature is lower than 30 °C, less water evaporates, the water replenishment accuracy exceeds the accuracy of the water replenishment machine, the system temperature fluctuates less, and the degree of denaturation of the polishing slurry is smaller. Secondly, when the temperature of STPS is higher than 50 °C, the water in STPS would evaporate rapidly, resulting in solidification and drying of the sample after the experiment, and even potentially impacting on the experimental equipment, making the obtained rheological data lose representativeness and repeatability. Therefore, this article selects 5 temperatures for the temperature study, namely 30 °C, 35 °C, 40 °C, 45 °C, and 50 °C, to analyze the effect of temperature on STP performance, as shown in Table 1. In order to obtain clearer detailed images of the STPS and a more balanced contrast between light and dark, this paper selected 1000 FPS as the frame rate for high-speed camera observation analysis. In order to eliminate the interference of temperature changes on MRR, quartz glass with low linear expansion coefficient, high temperature resistance and excellent chemical stability was selected as the workpiece material. Its physical parameters are shown in Table 2.

### 2.2. Measurement Method

The shear-thickening behaviors of STPS were observed by a high-speed camera (Keyence VW-9000, Osaka, Japan), and its rheological curve was measured using a Thermo Fisher MARS40 rotational rheometer (Berlin, Germany). The polishing force was measured using a JNNT-F torque sensor produced by Zhongwan Jinnuo (Fuyang, China), with a range of 50 N∙m and a resolution of 0.01 N∙m. Each group of experiments was measured three times, and the average value was taken. The actual polishing force was calculated based on the relationship between the force arm and the torque.

In the experiment, the workpiece inclination angle was *θ*, and the polishing force consisted of the pressure resistance *F_d_* and the friction resistance *F_f_*. The pressure resistance *F_d_* was along the flow direction of the slurry, while the friction resistance *F_f_* was oriented tangentially to the interface between the slurry and the cylindrical surface.

(1) $$T=L_{1}\cdot \left(F_{f}+F_{d}\right)\cdot \cos\theta$$

Among them, L_1_ is the lever arm.

The mass removed during polishing was measured using a precision balance (MSA225S-CE, Sartorius, Göttingen, Germany) with an accuracy of 0.01 mg and each measurement was repeated three times to obtain the average value. MRR is determined from the mass difference of the workpiece before and after the test and is calculated using the following formula:

(2) $$H_{MRR}=\frac{10^{7}{∆}_{m}}{\rho st}$$

where ∆_m_ is the mass difference before and after polishing; ρ is the workpiece density, in g/cm^3^; S is the workpiece area, in cm^2^; t is the polishing time, in min.

**Figure 4:**
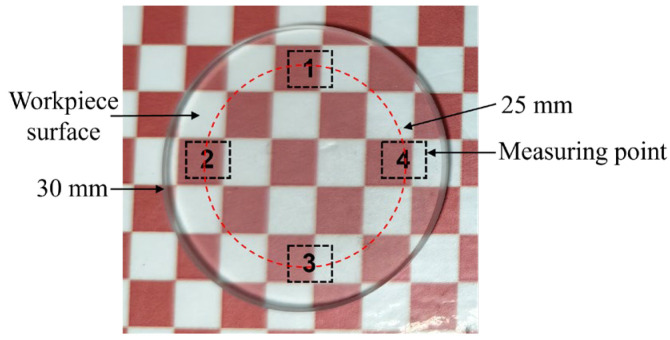
Workpiece measurement area.

Surface roughness and topography at different positions were analyzed using a white light interferometer (Super View W1, Lemayi Company, Shenzhen, China) and an optical microscope (VHX-7000, Keyence, Osaka, Japan). The sampling positions on the workpiece are shown in Figure 4, and the average of the measured values was calculated.

## 3. Experimental Results and Analysis

### 3.1. Rheological Properties of STPS at Different Temperatures

**Figure 5:**
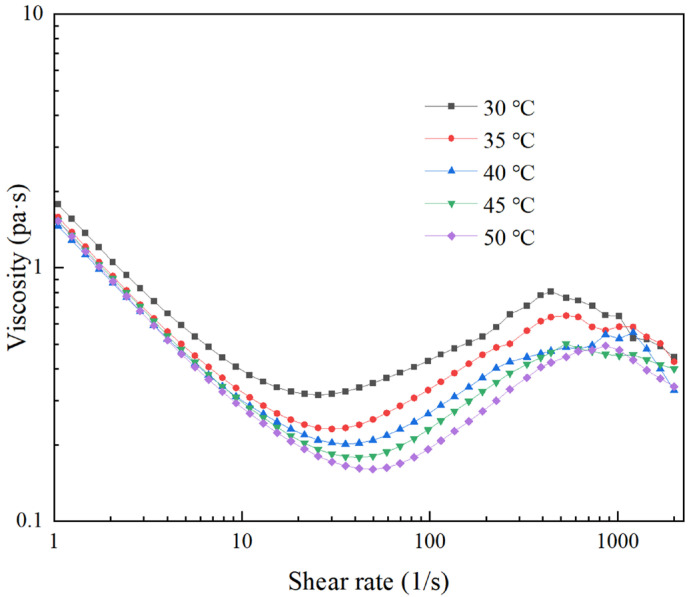
Rheological curves at different temperatures.

As shown in Figure 5, the rheological curves of the STPS at different temperatures are presented. It can be seen that the rheological properties of the STPS show three typical stages as the shear rate changes: shear thinning, shear thickening, and shear thinning. With increasing temperature, the shear-thinning phase extends, and the critical shear rate for shear thickening shifts upward, indicating that a higher shear rate is required to induce the shear-thickening effect. This phenomenon may be attributed to the enhanced thermal motion of particles at elevated temperatures, which increases disordered collisions between solid-phase particles and abrasives, thereby hindering particle cluster formation. Only when the shear rate further increases do the ordered motion of particles [26] and the friction frequency intensify, leading to increased internal resistance and the onset of shear thickening, thereby shifting the critical point to a higher shear rate.

During the shear-thickening stage, the critical shear rate of the slurry decreases at lower temperatures, while its viscosity rises more rapidly. This occurs because lower temperatures weaken the thermal motion of particles, facilitating the formation of stable particle clusters at lower shear rates [27,28,29], thereby increasing slurry viscosity. When the shear rate continues to increase and reaches the critical stress, the particle clusters may break due to the internal shear force, or gaps may form between the shear-thickening layers, resulting in a loose local structure and a decrease in friction, which in turn causes the slurry to enter the shear-thinning stage again.

Temperature has the following effects on the overall rheological properties of STPS. Higher temperatures delay the onset of shear thickening and weaken its viscosity-increasing effect, causing thickening to occur at higher shear rates. At lower temperatures, the slurry tends to form a denser shear-thickening structure. Consequently, the critical stress is reached at a lower shear rate, causing the structure to collapse and leading to the early onset of the shear-thinning stage.

### 3.2. Effect of Temperature on Shear-Thickening Rheological Behavior

**Figure 6:**
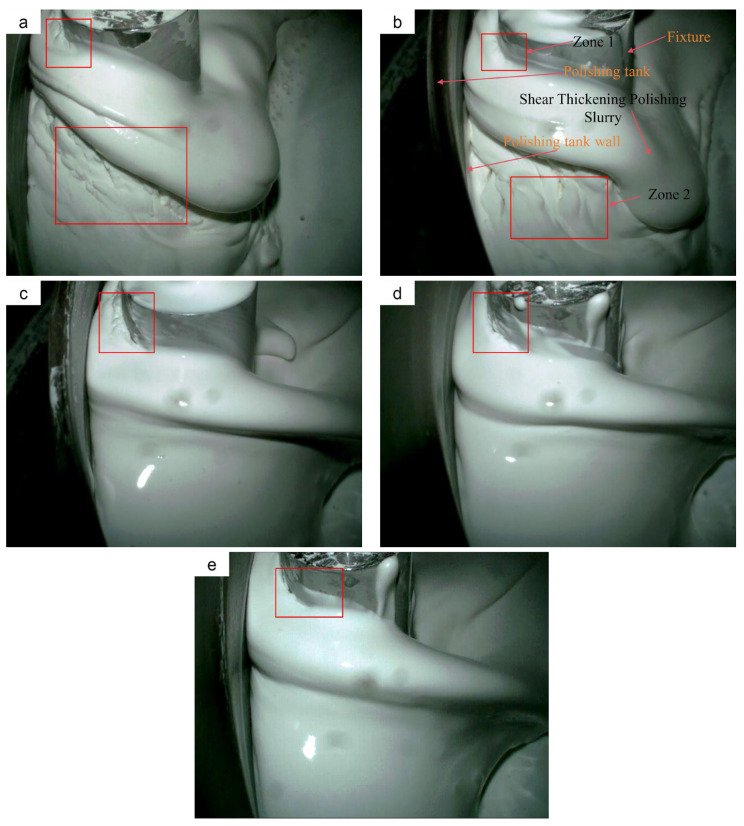
Shear-thickening phenomena observed through a high-speed camera under different temperatures: (**a**) 30 °C, (**b**) 35 °C, (**c**) 40 °C, (**d**) 45 °C, (**e**) 50 °C.

As shown in Figure 6, the image shows the changes in the slurry morphology when the STPS impacts the front of fixture. The front perspective was chosen to more intuitively capture the typical thickening characteristics of the STPS. Each set of experiments was repeated three times to ensure the repeatability and stability of the results. The red box in the figure marks the shear-thickening layer, whose structure is obviously different from the surrounding slurry. The layer presents a regular convex peak structure with obvious intervals between the peaks, forming a crack-like morphology. There are two zones at 30 °C that are obviously different from the surrounding STPS, namely Zone 1 and Zone 2, which show obvious shear-thickening behavior. As the temperature exceeds 40 °C, the thickening characteristics of Zone 2 gradually disappear, while Zone 1, close to the top end of the fixture, still maintains a certain shear-thickening behavior. It indicates that the slurry presents different rheological properties under different temperature which are consistent with the rheometer testing results. In theory, the experimental slurry is in a state of continuous motion and has compressibility and viscosity, and cracks should not occur during the flow process. However, the wavy cracks recorded by the high-speed camera indicate that, under specific conditions, the STPS transiently exhibits semi-solid or solid-like properties. This phenomenon further confirms the key mechanism of shear thickening: when the shear rate surpasses the critical rate, local particle clusters aggregate into a dense structure, causing the slurry to exhibit solid-like behavior. As shear stress increases within the thickening layer, the particles surpass their load-bearing limit, leading to the breakdown of the solid structure and the occurrence of interlayer slip. As the crack propagates, the particle clusters gradually disintegrate, causing the slurry to enter the shear-thinning stage, accompanied by a corresponding decrease in viscosity.

The experimental images provide an intuitive demonstration of the temperature effect on the shear-thickening phenomenon. With increasing temperature from 30 °C to 50 °C, the solid wave cracks progressively diminish, and their distribution becomes confined to the fixture interface, indicating that the shear-thickening effect is weakened. As temperature increases, the thermal motion of particles intensifies, reducing the stability of particle clusters. Additionally, solid-phase particles may weaken their binding force on abrasive particles due to thermal expansion or surface modifications, thereby diminishing the shear-thickening strength. As shown in Figure 6, at higher temperatures, the constraint force exerted by particle clusters on abrasive and solid-phase particles weakens, leading to their dispersion. Under identical cutting conditions, particle clusters at elevated temperatures exhibit a reduced capacity to withstand cutting reaction forces, making them more susceptible to deformation and disintegration, thereby diminishing the material removal efficiency of STP.

With further heating, the thickness of the shear-thickening layer decreases, and the wavy cracks nearly disappear. This trend aligns with the rheological curve results, confirming that the viscosity of the slurry decreases as the temperature increases. In comparison to the slurry shear-thickening layer, the solid thickening layer experiences smaller deformation and shorter contact time when impacting the workpiece surface under the same momentum. Therefore, an increase in temperature reduces the formation of the solid shear-thickening layer, diminishes the polishing force on the workpiece, and lowers MRR. In practical applications, controlling temperature is crucial for maintaining the stability of the STPS and ensuring effective polishing.

### 3.3. Polishing Force on the Workpiece at Different Temperatures

**Figure 7:**
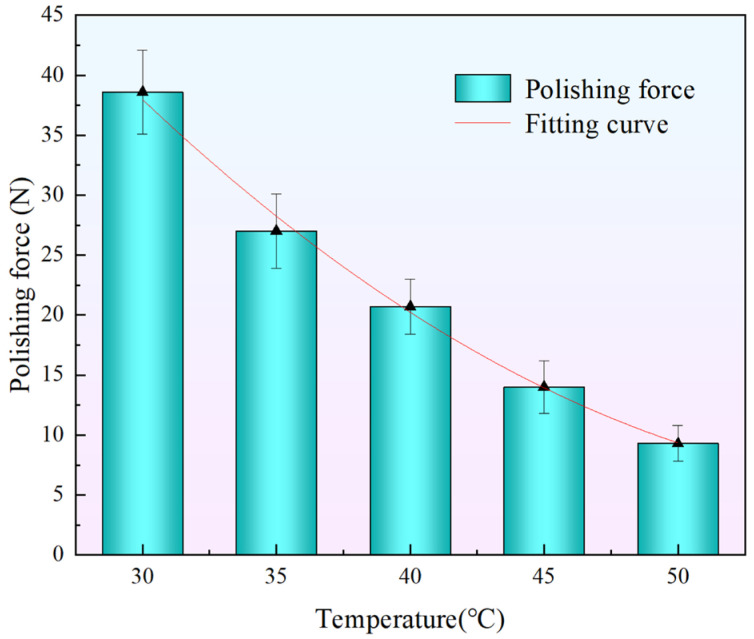
Actual values and fitting curves of temperature and polishing force.

As shown in Figure 7, the polishing force on the workpiece varies significantly under different temperature conditions. As the temperature increases from 30 °C to 50 °C, the initial polishing force exerted by the STPS on the workpiece gradually decreases. Experimental data show that as the temperature increases from 30 °C to 40 °C, the polishing force decreases from 38.6 N to 20.7 N, a reduction of 46.4%. With a further increase to 50 °C, the polishing force decreases to 9.3 N, which is 55.1% lower than at 40 °C.

In general, temperature has a significant impact on the workpiece polishing force, with a trend of rapid decline followed by a gradual slowdown. Specifically, the polishing force specifically drops off significantly between 30 °C and 40 °C, suggesting that the shear-thickening effect is strongly suppressed in this temperature range. From 40 °C to 50 °C, the polishing force continues to decrease, but at a slower rate, suggesting that the effect of temperature on shear thickening stabilizes at higher temperatures.

This trend is closely related to the rheological properties of the STPS. The shear-thickening phenomenon is primarily driven by the aggregation of microscopic particles, and an increase in temperature enhances the thermal motion of the particles, reducing the viscosity of the slurry and weakening the interactions between the particles.

As shown in Figure 7, the fitting curve of polishing force versus temperature is presented. Under the experimental parameter conditions, the relationship between the two is approximately fitted into this mathematical relationship. The root mean square error of the fitting curve is 0.637, and the determination coefficient R^2^ is 0.9962, indicating that the model can well describe the relationship between temperature and polishing force.

(3) $$F=138.54-4.495T+0.0383T^{2}$$

**Figure 8:**
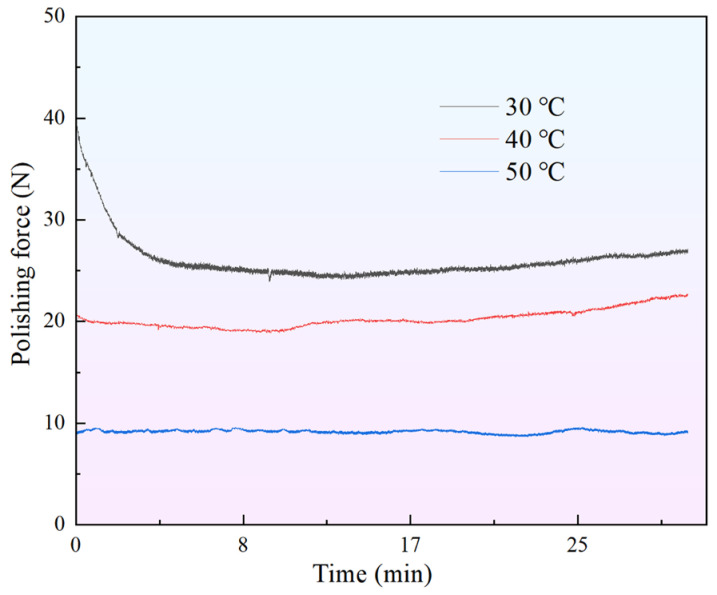
Polishing force at different temperatures.

The changes in the polishing force of the STPS on the workpiece within 30 min at different temperatures are shown in Figure 8. Since the experimental temperature exceeds room temperature, water is added to counteract the effects of evaporation and maintain a constant mass fraction of water molecules, ensuring that temperature remains the only variable. At 30 °C, the polishing force decreases from an initial 38 N to 25 N and stabilizes. This may be due to the uniform distribution of solid-phase and abrasive particles in the STPS during the initial stage, which facilitates particle cluster formation and enhances the shear-thickening effect. However, over time, solid-phase and abrasive particles settle under the influence of centrifugal force and gravity during circular motion, reducing their content in the upper layer, increasing the proportion of water molecules, decreasing the likelihood of particle cluster formation, weakening the shear-thickening effect, and leading to a reduction in polishing force. Since the workpiece is statically immersed in the rotating STPS, the system can be treated as a stirrer. During the interaction of the STPS with the workpiece, the slurry surges, and the settled solid-phase and abrasive particles are stirred and recirculated. When the recirculated and sedimented particles reach dynamic equilibrium, the polishing force stabilizes and no longer decreases. This stabilization process demonstrates that, under specific temperature and motion conditions, the distribution of solid-phase particles in the STPS can reach a dynamic steady state, resulting in a relatively constant polishing effect.

When the temperature rises to 40 °C and 50 °C, the decrease in the initial polishing force becomes less significant or even disappears. There are two possible reasons: firstly, the temperature increase enhances the thermal motion of the particles, improving the ability of the solid-phase and abrasive particles to resist gravity and centrifugal force, thereby slowing down sedimentation. Secondly, the temperature increase may cause the polyhydroxy polymer in the STPS to denature, enhancing water absorption capacity, expanding volume, reducing the proportion of exposed water molecules, and increasing the adhesion between solid-phase particles, which enhances their ability to wrap abrasive particles. In addition, as the solid particles absorb water and swell, their total mass fraction increases, making it difficult for them to separate from the water molecules and settle or stratify.

In general, temperature affects both the shear-thickening effect and the stability of the polishing force by altering the motion characteristics of the solid particles, the slurry’s viscosity, and its water absorption capacity. Experimental results indicate that at lower temperatures, the polishing force of the STPS is more susceptible to particle sedimentation, initially decreasing before stabilizing. In contrast, at higher temperatures, the obstruction of particle sedimentation leads to a more stable shear-thickening effect, maintaining a relatively constant polishing force for a longer period.

### 3.4. MRR of Workpiece at Different Temperatures

**Figure 9:**
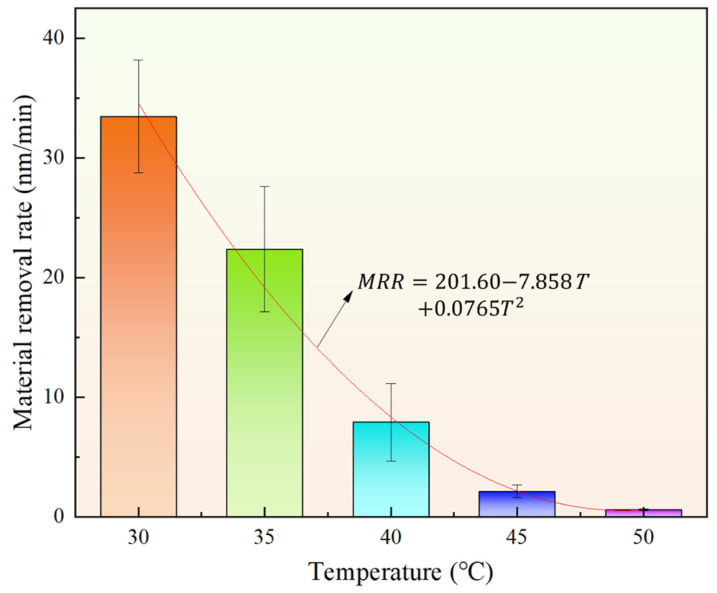
MRR at different temperatures.

Figure 9 illustrates the changes in MRR of the workpiece at various temperatures. As the temperature increases, MRR decreases markedly. It decreases from 33.5 nm/min at 30 °C to 7.9 nm/min at 40 °C. As the temperature increases further to 50 °C, MRR decreases to just 0.6 nm/min. This demonstrates that temperature has a substantial effect on MRR. The root mean square error of the fitting curve in Figure 9 is 1.740, and the determination coefficient R^2^ is 0.9816, indicating that the model can well describe the relationship between temperature and MRR.

**Figure 10:**
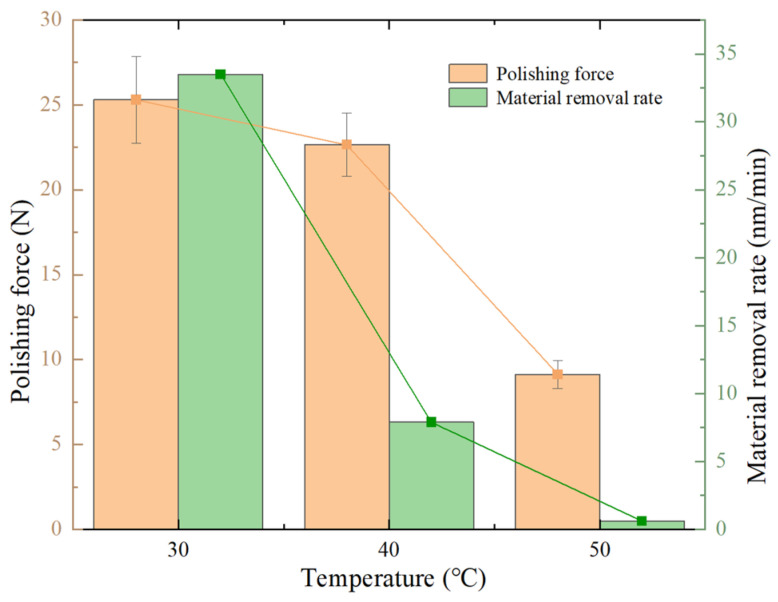
Polishing force and MRR at different temperatures.

Three trials were conducted for each group of the same temperature parameters to obtain the average force value for the same parameters, where the selected temperatures were 30 °C, 40 °C, and 50 °C. The effect of temperature on the average polishing force and MRR is shown in the same figure, as shown in Figure 10. From the figure, it is evident that when the temperature increases from 30 °C to 40 °C, the polishing force decreases from 25.3 N to 22.6 N, a reduction of 10.6%. However, the reduction in the MRR is much greater than that of the polishing force, specifically decreasing from 33.5 nm/min to 7.9 nm/min, a reduction of 75.5%. This result indicates that the increase in temperature not only reduces the polishing force on the workpiece but also significantly alters other factors affecting MRR, leading to a much more pronounced reduction in MRR than in polishing force.

**Figure 11:**
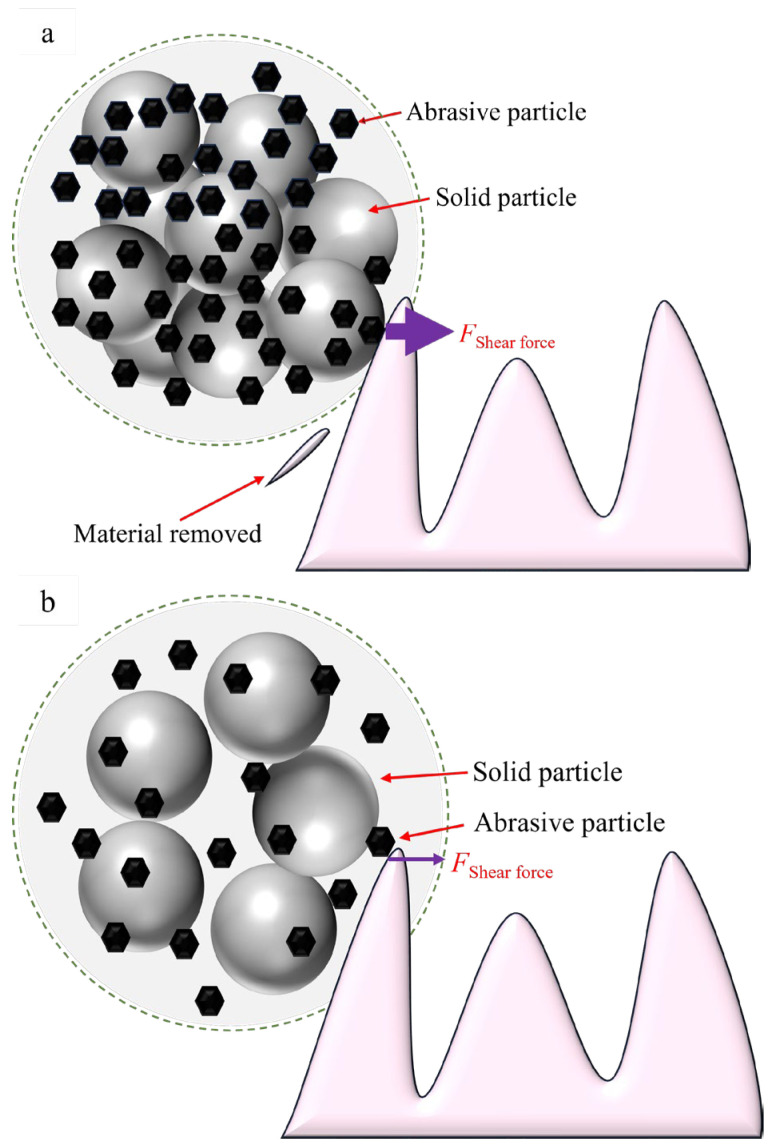
Micro-cutting of particle clusters at different temperatures: (**a**) lower temperature, (**b**) higher temperature.

The increase in temperature may lead to a decrease in MRR of the workpiece, primarily due to its effect on the stability of the particle clusters. As shown in Figure 11, at lower temperatures, the binding force between the solid-phase particles and abrasive particles is strong, resulting in a compact particle cluster structure. During the polishing process, these particle clusters exert a significant cutting force on the workpiece surface due to the extrusion impact of their own momentum and the STPS. Since the solid-phase particles firmly adsorb the abrasive particles, the entire particle cluster structure acts as a whole to effectively cut the microscopic rough peaks on the workpiece surface, thereby facilitating material removal. However, as the temperature increases, the solid-phase particles deform, leading to a looser particle cluster structure, as shown in Figure 11a. Higher temperature reduces the holding force of particle clusters on abrasive particles. When impacting the workpiece surface, the particle cluster disintegrates due to the reaction force, losing its ability to control the abrasive particles effectively, which reduces the material removal efficiency. As shown in Figure 11b, the polishing force’s reaction force causes the particle cluster to disintegrate. This change indicates that under high-temperature conditions, the rheological properties of the STPS alter, making the particle clusters less stable during the cutting process compared to low temperatures, thereby affecting the transmission of cutting force.

Li [30] et al. proposed an adaptive shear gradient thickening polishing method in the process of improving the surface accuracy of lithium niobate crystals and inhibiting surface damage. The experimental results show that the increase in temperature will cause the hydrogen bonds between the amide groups and water molecules in the copolymer to gradually break, causing the copolymer to undergo thermally induced phase separation. At the same time, the increase in temperature during processing weakens the ability of stress to maintain viscosity and frictional contact, destroying the network microstructure in the system. This phenomenon is consistent with the conclusion of this paper: the increase in temperature weakens the constraint effect of particle clusters on abrasive particles, reducing the effective polishing force and, thus, leading to a decrease in MRR.

In summary, the increase in temperature significantly impacts the polishing effect of the STPS, primarily by weakening the stability of the particle clusters, which reduces MRR. To ensure optimal polishing performance under high temperature conditions, adjustments to the formulation of the STPS may be necessary. For instance, optimizing the composition of the solid-phase particles to enhance their stability at high temperatures or adjusting the rheological properties of the slurry to ensure the shear-thickening effect remains effective at elevated temperatures.

### 3.5. Surface Roughness and Morphology at Different Temperatures

**Figure 12:**
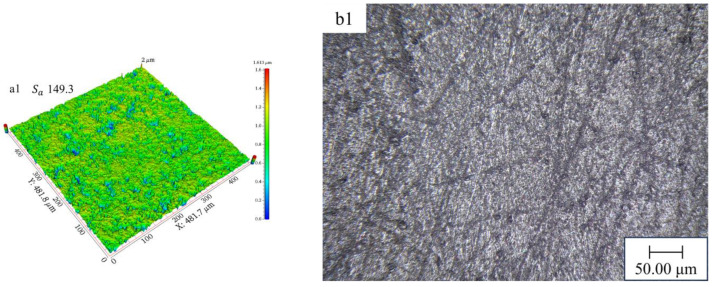

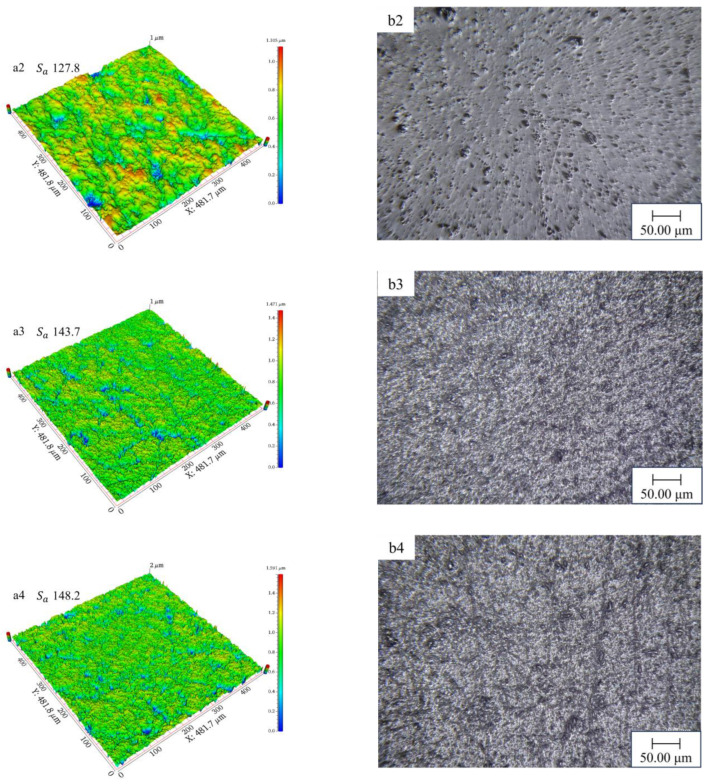
Effect of temperature on workpiece surface: (**a1**) initial surface roughness, (**b1**) initial surface morphology, (**a2**) surface roughness at 30 °C, (**b2**) surface morphology at 30 °C, (**a3**) surface roughness at 40 °C, (**b3**) surface morphology at 40 °C, (**a4**) surface roughness at 50 °C, and (**b4**) surface morphology at 50 °C.

As MRR decreases, the amount of roughness peak removal on the workpiece surface is reduced, and then the change in surface morphology is also decreases accordingly. In order to show that the change of MRR in the experiment affects the degradation value of the surface morphology, a white light interferometer (Super View W1) and a light microscope (VHX-7000) are used to observe the changes in the surface morphology of the workpiece. In the experiment, the surface roughness and morphology of the workpiece after STPS polishing for 30 min at different temperatures are shown in Figure 12. The experimental results indicate that the change in surface roughness follows a trend consistent with MRR. As the temperature increases from 30 °C to 40 °C and 50 °C, MRR decreases from 33.5 nm/min to 7.9 nm/min and 0.9 nm/min, and the surface roughness Sa of the workpiece also decreases, from 21.5 nm to 5.6 nm and 1.1 nm, respectively. This demonstrates that the increase in temperature weakens the holding force of the particle clusters on the abrasive and solid particles during the shear-thickening process, thus affecting the polishing effect.

**Figure 13:**
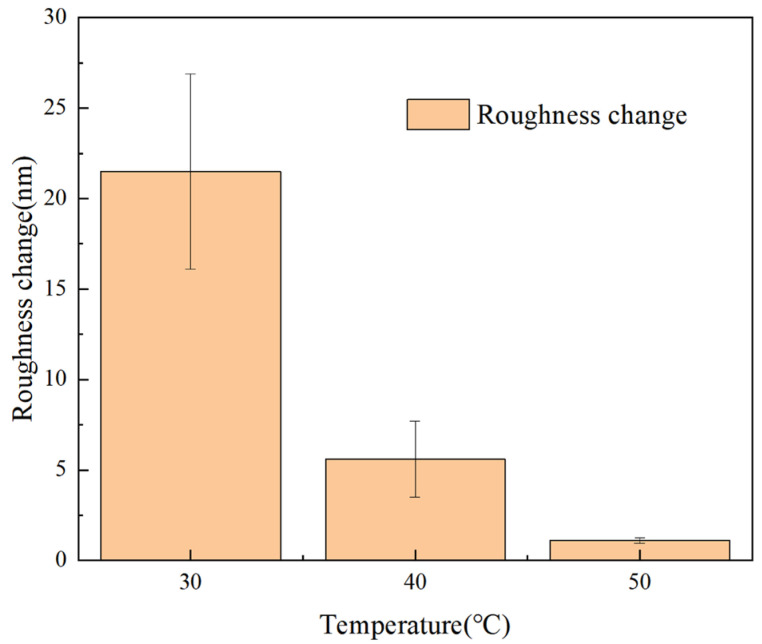
Effect of temperature on surface roughness.

Further analysis of the surface morphology changes reveals that the alteration in the surface morphology of the workpiece gradually decreases with increasing temperature. At 50 °C, the surface morphology of the workpiece is nearly at its initial state, indicating that the removal capacity of STP is significantly reduced under high-temperature conditions. This further confirms that the weakening of the shear-thickening effect with rising temperature, along with the decreased stability of particle clusters, leads to a substantial reduction in polishing effectiveness. Figure 13 illustrates the effect of temperature on surface roughness.

Overall, STPS exhibits better polishing performance at lower temperatures, effectively removing material and reducing the surface roughness of the workpiece. Under high-temperature conditions, the stability of the STPS is reduced, weakening the effectiveness of particle clusters, and resulting in a significant decrease in MRR and polishing effectiveness. Therefore, temperature significantly affects the polishing performance of STPS, and higher temperatures are unfavorable for reducing the surface roughness of the workpiece.

## 4. Conclusions

This study experimentally measures the rheological curves of STPS at various temperatures and observes the corresponding shear-thickening behavior. By analyzing the polishing force, MRR, and changes in the surface morphology of the workpiece during polishing, the following conclusions are drawn:

(1) As the temperature increases from 30 °C to 50 °C, the peak viscosity of the STPS decreases from 0.81 Pa·s to 0.49 Pa·s. An increase in temperature prolongs the shear-thinning stage and shifts the critical shear rate for shear thickening to a higher value, requiring a higher shear rate to trigger the thickening effect.
(2) In the high-speed video of the workpiece subjected to a frontal impact, the STPS exhibits solid-like properties in the local thickening area, and the thickening layer forms wavy cracks under the action of shear stress and impact force. As the temperature rises, the thermal motion of the particles increases, and the stability of the particle clusters may decrease, which is manifested as a weakening of the shear-thickening effect in the image and an increase in fluidity. When the temperature increases from 30 °C to 50 °C, the wavy cracks decrease and almost disappear, which may be the reason for the decrease in polishing force and MRR.
(3) As the temperature of the STPS rises, the initial polishing force on the workpiece decreases. The decrease is initially rapid, followed by a gradual slowing down. The polishing force decreases by 46.4% from 30 °C to 40 °C and by 55.1% from 40 °C to 50 °C, exhibiting an exponential decline.
(4) When the temperature increases from 30 °C to 50 °C, the average polishing force decreases from 25.3 N to 22.6 N, a 10.6% reduction, while MRR decreases from 33.5 nm/min to 7.9 nm/min, a 75.5% reduction. This is due to the weakening of the holding force between the particle clusters and the abrasive and solid particles at higher temperatures, a phenomenon that makes them more likely to disintegrate upon impact with the workpiece, preventing effective material removal. Furthermore, the reduction in surface roughness becomes more pronounced with increasing temperature, with the final surface morphology approaching its initial state.

## 5. Review and Outlook

From a mechanistic perspective, the explanation in this paper that temperature increases lead to a decrease in MRR remains speculative, and more direct experimental evidence is needed to verify the effect of temperature on the ability of particle clusters to hold abrasives. This would help clarify the underlying mechanism of action influencing MRR. In terms of experimental data, high-speed camera observation is an indirect means of evidence. In the future, image algorithms will be combined with higher-resolution imaging technology to further explore the dynamic behavior of microscopic particles. This study provides an experimental foundation for understanding the effect of temperature on STP behavior and lays the groundwork for subsequent research into the coupling effects of multiple factors, such as temperature, force, and motion.

## Figures and Tables

**Table 1:** Experimental conditions.

Experiment Parameters	Values
Temperature (°C)	30, 35, 40, 45, 50
Shooting frame rate (FPS)	1000
Polishing tank diameter (mm)	400
Workpiece diameter (mm)	30
Abrasive	Al_2_O_3_
Abrasive concentration (%)	7
Speed (rpm)	60
Polishing time (min)	30

**Table 2:** Physical parameters of quartz glass.

Quartz Glass Parameters	Values
Density (g/cm^3^)	2.45
Hardness (Mohs)	7.5
Linear expansion coefficient (K^−1^)	5.4 × 10^−7^
Initial roughness (nm)	150 ± 10
Size (mm)	30 × 2

## Data Availability

The original contributions presented in this study are included in the article. Further inquiries can be directed to the corresponding authors.
